# The effect of mobile-based training on maternal breastfeeding self-efficacy: a randomized clinical trial

**DOI:** 10.4314/ahs.v22i3.69

**Published:** 2022-09

**Authors:** Aazam Seddighi, Zahra Bostani Khalesi, Soheila Majidi

**Affiliations:** 1 Student Research Committee, school of nursing and midwifery, Guilan University of Medical Sciences, Rasht, Iran; 2 Social Determinants of Health Research Center, Guilan University of Medical Sciences, Rasht, Iran; 3 Department of Midwifery and Reproductive Health, Nursing and Midwifery School, Guilan University of Medical Sciences, Langroud, Iran

**Keywords:** Self-efficacy, breastfeeding, cell phone, mobile applications, education

## Abstract

**Background:**

The Aim of this study is to determine the effect of mobile-based training on maternal breastfeeding self-efficacy.

**Materials and Methods:**

This trial was conducted from November May to December 2020 on 198 women referring to healthcare centers in Guilan, Iran. The samples of this study were selected using the convenience sampling method, and random block sampling was used for the allocation of groups. The data collection tool was a two-part questionnaire including questions about demographic data and Dennis's self-efficacy. The questionnaires were completed before and 8 weeks after the intervention in both groups.

**Results:**

The mean and standard deviation of self-efficacy before the education in the experiment and control group were 48.26+ 6.49 and 49.11 + 7.36, respectively. After the education, the experimental group was 53.78 + 12.61 and control group was 41.90 + 17.98. The difference between the pretest and posttest scores indicated that the breastfeeding educational intervention increased the women's self-efficacy in breastfeeding (p<0/001).

**Conclusion:**

The results showed that mobile-based training could improve maternal breastfeeding self-efficacy. It is therefore recommended, this training program as an available and convenient method to improve breastfeeding self-efficacy.

## Introduction

Breastfeeding is the natural and safe way of feeding infants and is essential to ensure infant's growth and development^1^. Because it meets infant needs and establishes a deeper emotional relationship between mother and child^2^. Breastfeeding is an effective way and beautiful process that helps create intimacy and bonding between mom and baby^3^. Human milk is as a first vaccination that protects infants against a wide variety of infections; diarrhea; respiratory infection, and sudden death^4^.

Although most mothers Consider breastfeeding to be the best nutrition for their baby until they are 2 years old, they mostly fail to continue it in the first week after birth^5^. One of the causes of failure to continue breastfeeding is the low self-efficacy of the mother in breastfeeding^6^.

Self-efficacy is an important psychological and motivational factor in breastfeeding ability^7^. According to Albert Bandura's theory of social cognition, Self-efficacy consists of the person's beliefs and ideas toward doing responsibilities^8^. Many factors affect breastfeeding self-efficacy, including Age, previous experience and mother's attitude, verbal encouragement from family and friends, peer breastfeeding education^9^. Nipple sores and fissures, flat or sunken nipples, breastfeeding pain, stress and anxiety, low birth weight, premature baby, Cesarean delivery and Working mother have a negative effect on breastfeeding self-efficacy^11^. Also, many studies have confirmed the positive effect of education on breastfeeding self-efficacy. However, one of the most important factors in the effectiveness of educational programs is choosing the most appropriate method and educational media to transfer information to the audience^11^.

One of the modern educational methods, which are approved by researchers, is using the developed electronic technology because the traditional methods no longer meet the learner's needs and do not give them enough information^12^. Following the changing expectations of society, people are looking for a training strategy that is personal, based on competency, and it can happen at any anytime, anywhere^8^. The need for such a strategy forces designers to try to create effective and efficient systems for the development of electronic learning^13^. Computer-based training has attracted increasing attention from researchers in the recent decade^14^. With the development of the use of computers, research more was performed on computer-based training^15^. This progress helped to access the global goal of education and learning for all^16^. Mobile phones also such as computers were become a learning tool with great potential^17^. Learning through mobile apps using mobile devices to obtain learning materials so-called mobile learning^18^. With mobile learning, there is no limitation to learn and people can be educated without a presence in the classroom^16^. Another advantage is ease-of-use and flexibility, full-time learning, reduces the pressure for attending courses, and lowering the cost of education^19^. Since many mothers have expressed a need for suitable information on breastfeeding, using an educational method to achieve this aim seems necessary. Therefore, this study was conducted to effect of mobile-based training on maternal breastfeeding self-efficacy.

## Material & Method

This trial was conducted from November May to December 2020 on 198 women referring to healthcare centers in Guilan, Iran. The samples of this study were selected using the convenience sampling method, and random block sampling was used for the allocation of groups.

The sample size was estimated at 74 eligible women per group based on a study by Wu DS et al., using the sample size formula with a 95% confidence and the power of 90% (, and considering a 25% attrition rate.

According to the following formula:

$$\begin{array}{c} n=\frac{{\left(z_{1-\frac{\alpha }{2}}+z_{1-\beta }\right)}^{2}\times \sigma^{2}}{d^{2}}\\ \sigma^{2}=2\times 7/4^{2}\times (1-0.5)=54/76\\ n=\frac{{\left(1/96+0/84\right)}^{2}\times 54/76}{2/4^{2}}=74 \end{array}$$

To find the final adjusted sample size, a non-response rate of 25%, the adjusted sample size will be:

$$\begin{array}{l} \text{n}f=\frac{\text{n}}{1-\text{f}}\\ \text{n}f=\frac{\text{74}}{0.75}=99 \end{array}$$

Participants were all the mothers who meet the inclusion criteria and referring for infants' to comprehensive health centers within 2–4 days after birth. Iranian nationality, ability to read and write, beginning breastfeeding the first 24 hours after birth, no history of underlying or chronic diseases, absence of addiction to alcohol or tobacco or drugs, lack of medical prohibition for breastfeeding, possessing mobile phone with android operating system compatible with intervention application, ability to work with and use mobile phone applications, no history of education or not being a graduate in medical fields. Exclusion criteria were refusal to participate in the study at any time.

Based on the proportional-stratified sampling method, the city was divided into 4 districts (i.e., Northern, Southern, Eastern, and Western districts). The affiliated Health centers of the Guilan University of medical sciences in Rasht were the data extract's centers. After calculating the number of samples in each district, a number of comprehensive health centers were randomly selected, and the researcher referred to them for selecting participants among mothers who were referrng to the centers to receive their postnatal care. Participants were selected using the convenient sampling method. 198 women, who were recruited to the study using the convenience sampling method, were randomly divided into the intervention (n = 99) and control (n = 99) groups. Assigning individuals to the groups was carried out through the block randomization method.

**Figure 1:**
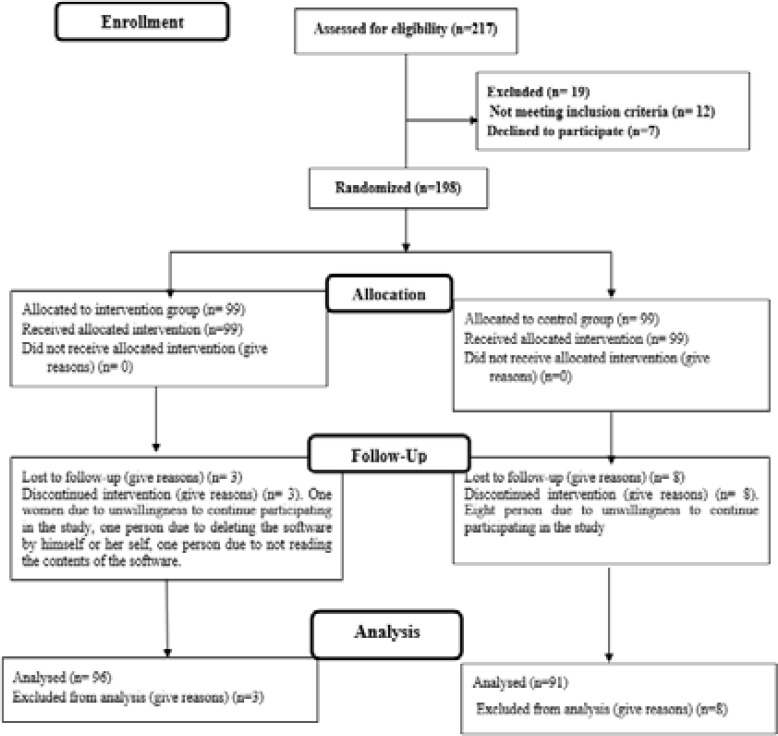
Consort flow diagram

The researcher gave participants a number from 1 to 198. By random allocation software, a table with 50 rows of blocks be designed and each block was named A and B. The numbers were placed in each house in order, after that, all numbers were placed in blocks. 50 Quadruple blocks were randomly selected. Random blocks were placed in sealed and locked envelopes and kept at selected health centers and no one knew their sequence. After starting the study, the blocks were randomly selected daily. Those in house A were placed in the test group and those in house B were placed in the control group (Figure1).

The data collection tool consisted of a demographic questionnaire and breastfeeding self-efficacy scale short form (BSES-SF). The BSES-SF is a 14-item instrument developed to measure breastfeeding confidence. All the items are preceded by the statement ‘I can always' and are anchored by a 5-point Likert-type scale, with 1=not at all confident and 5=always confident. All the items are presented positively and the scores are summed up to produce a final score ranging from 14 to 70, with the higher scores indicating better breastfeeding self-efficacy. The BSES-SF was designed in 1999^20^ and Arban et al^21^ confirmed its credibility, and its reliability was confirmed with the internal conformity of alpha Cronbach 91%. Cronbach's α of the BSES-SF in this study was 0.94.

The content of the mobile-based education app was prepared based on the items of the breastfeeding self-efficacy questionnaire from the breastfeeding promotion educational booklet by the Ministry of Health and Medical Education, which including the benefits, breast milk ingredients, patterns of daily duration and frequency of breastfeeding, common breastfeeding positions , use of a pacifier, signs of being full or hungry in infants, signs of baby's hunger and fullness, different positions of mother in breastfeeding, Identify the indicators of adequacy of breast milk, proper storage and preparation of breast milk, common causes of a breast-feeding strike, cracked nipples, breast engorgement, premature and low weight infants, mother's common cold, taking medications while breastfeeding, infant's diarrhea, ways to way to increase and continue milk supply, supplements to increase milk supply and disadvantages of powdered milk. Mothers the intervention group received the smartphone-based app besides routine care. Mothers were taught how to use the program face-to-face session. Every week for eight consecutive weeks, notifications using SMS were sent to the participants to remind them to use the app. During the app usage period (8 weeks), the participants were supported in terms of how to use the program. To provide support, the researcher's telephone number was provided to the participants for contact purposes, if needed. The control group receiving routine care. After 8 weeks, the breastfeeding self-efficacy questionnaire was completed in both groups.

In the end, out of 198 samples, 3 of them in the experimental group(1 for unwillingness and 2 for not studying the app materials), and 8 people in the control group (3 for unwillingness and 5 due to call failure during 8 weeks of study exited from the study)were excluded from the study. Statistical analysis was performed using descriptive statistics. Shapiro-Wilk and Kolmogorov Smirnov tests were used to evaluate the normality of the data. In order to compare the inferential data in groups before and after the intervention, data were analyzed using spss16 software under version 16 and Mann-Whitney u, Wilcoxon, Kruskal-Wallis, and also chi-square and multiple regression tests. And significance level was considered P<0.05. This study was approved by the ethics committee of Guilan University of Medical Sciences with the code number of “IR.GUMS.REC.1398.106” and clinical trial code IRCT20190615043895N1.

## Result

Based on the findings of the demographic questionnaire, the mean age of participants was 29.58 ± 5.32, most of them (43.32%) had a university education, and homemaker (75.4 %), and their type of delivery was cesarean delivery (72.73%). Most of them had a monthly income of twenty million Iranian Rial and above (36.90%) (Table 1).

**Table 1:** Demographic characteristics of participants in the experimental and control groups

variable		Experimental Group N = 96	Control Group N = 91	P value
Mother's age	Mean	29.8±5.06	29.3±5.6	0.689
Mother's education	primary	1.04	4.4	0.361
	elementary	4.17	8.79	
	secondary	7.29	5.49	
	diploma	40.62	41.76	
	University education	46.88	39.56	
Mother's job	housewife	68.75	82.4	0.069
	Working in health centers	3.13	3.3	
	Working in other fields	28.12	14.3	
Type of delivery	Normal Vaginal Delivery	29.17	25.27	0.55
	Cesarean delivery	70.83	74.73	
Number of children	First child	64.6	57.1	0.386
	Second child	30.2	33	
	Third child and more	5.2	9.9	
Family income	One million and less	5.21	3.30	0.176
	One to one and half million	22.92	29.67	
	One and half to two million	28.12	37.36	
	Two million and more	43.75	29.67	

*Significance Level P<0.05

The breastfeeding self-efficacy score before the intervention was 49.11+7.36 and 48.26 ± 6.49 in the control and experimental groups, respectively. After the intervention, this score was 41.90 ± 17.98 in the control group and 53.78 ± 12.61 in the experimental group (Table 2).

**Table 2:** Comparison of mean breastfeeding self-efficacy scores in participants, before and after intervention in experiment and control group

Breastfeeding self-efficacy		Mean	Std. Deviation	Mean difference	SD difference	P value
Experiment	Before intervention	48.26	6.49	5.52	12.35	0.001>*
	After intervention	53.78	12.61			
Control	Before intervention	49.11	7.36	-7.21	16.82	0.024*
	After intervention	41.90	17.98			

* Significance Level P<0.05

The difference between the pretest and posttest scores indicated that the breastfeeding educational intervention increased the women's self-efficacy in breastfeeding (p<0/001).

## Discussion

The results showed that intervention with the use of mobile phone app is effective in promoting breastfeeding self-efficacy. The participants improved their breastfeeding self-efficacy through the information provided in the breastfeeding education. This improvement was shown by the increase in the BSES-SF instrument's posttest scores after completing the breastfeeding education. Thus, the null hypothesis was accepted. The findings are related to Bandura's social cognitive theory that an individual with a high sense of self-efficacy is likely to anticipate positive performances^22^. Bandura's social cognitive theory was relevant to this study because the women could access breastfeeding education that contained information on solutions to overcome breastfeeding problems for repeated use whenever desired. Previous research has found higher breastfeeding knowledge to positively affect both breastfeeding outcomes and breastfeeding intention^1^, ^3^, ^5^. In this study, most of the mothers (29.41%) had referred to comprehensive health centers to get information on breastfeeding. 27.27% of mothers received no education on breastfeeding during pregnancy. According to the results of Behzadifar's research, the most sources of information about child nutrition were health care, followed by radio, television, and books^23^. The study of Khayyati also shows that 51.6% of mothers used health center information while only 2.5 % used media as the most important resource of information in breastfeeding^24^. In the study of Khosravi et al, the main source of information was family, friends, and acquaintances^25^.

Also in the study of Poorahmad et al, the biggest resource of information were friends and acquaintances (47%)^11^. In the study of Mahmuodi et al, the main source of information was the internet and then interaction with friends^26^. In the study by Gudarzi et al.^5^, peer education was effective to promote breastfeeding self-efficacy. In the study, a significant relationship was found between breastfeeding self-efficacy and type of delivery, which is consistent with the results of some studies. In Nursan et al.^27^ study, although the breastfeeding self-efficacy was higher in mothers who had a cesarean section, no significant relationship was observed between breastfeeding self-efficacy and type of delivery. The results of Tokat et al. and Poorshaban et al. indicate a significant relationship between breastfeeding self-efficacy and type of delivery and mothers with a normal delivery had a higher self-efficacy than mothers with cesarean section^9^, ^28^. The study of Ahmadi et al showed that there was a significant difference between breastfeeding self-efficacy of women with normal delivery and cesarean section and acknowledged that women with normal delivery had higher breastfeeding self-efficacy compared to women with cesarean delivery^29^. The number of living children was positively correlated with higher breastfeeding self-efficacy. This is consistent with previous studies^30^. Similarly, Hinic found the number of livng children to be a predictor of breastfeeding self-efficacy in the immediate postpartum period among a sample of mixed primiparous and multiparous women^31^.

This study has a number of limitations. This study used a small sample. It was difficult to recruit subjects to this study; most of the women did not intend to breastfeed and therefore, did not meet the inclusion criteria.

## Conclusion

The results of the BSES-SF post-test scores showed that all of the participants had a high level of breastfeeding self-efficacy after accessing breastfeeding education.. Health providers could use this tool to improve their maternal breastfeeding self-efficacy. It is important that all breastfeeding mothers be followed up after delivery. After delivery, mothers often do not have the time to learn all they needed to know about breastfeeding. They often feel overwhelmed with the amount of material they are presented with and may feel tired or uncomfortable comprehending what they have been taught. They often leave the hospital not feeling confident in their abilities to breastfeed.
